# Financial risk of increasing the follow-up period of breast cancer treatment currently covered by the Social Protection System in Health in México

**DOI:** 10.1186/s12962-018-0094-y

**Published:** 2018-03-08

**Authors:** Román Rodríguez-Aguilar, José Antonio Marmolejo-Saucedo, Sonia Tavera-Martínez

**Affiliations:** 1 Facultad de Ingeniería, Universidad Anáhuac Mexico, Mexico, Mexico; 2 Facultad de Ingeniería, Universidad Panamericana, Augusto Rodin 498, 03920 Mexico, Ciudad de México Mexico; 3 Law School, Universidad Anáhuac Mexico, Mexico, Mexico

**Keywords:** Financial management, Financial risk, Micro simulation, Monte Carlo method, Breast cancer

## Abstract

**Background:**

The objective of this work is to estimate the financial impact of increasing the monitoring period for breast cancer, which is financed by the Sistema de Protección Social en Salud (SPSS—Social Protection System in Health).

**Methods:**

A micro-simulation model was developed to monitor a cohort of patients with breast cancer, and also an estimation was made on the probability of surviving the monitoring period financed by the SPSS. Using the Monte Carlo simulation, the maximum expected cost was estimated to broaden such monitoring. Morbimortality information of the Ministry of Health and cases of breast cancer treated by the SPSS were used.

**Results:**

Between 2013 and 2026, the financial resources to provide monitoring during 10 years to women diagnosed with breast cancer would reach up to $3607.40 million pesos on a base scenario, $4151.79 million pesos on the pessimistic scenario and $3414.85 million pesos on an optimistic scenario. In the base scenario, additional expenditure represents an annual increase of 9.1% of resources allocated to treating this disease, and 3.0% of the availability of the resources for the Fondo de Protección contra Gastos Catastróficos (FPGC—Fund for Protection against Catastrophic Expenditure).

**Conclusions:**

Increasing monitoring for patients with breast cancer would not represent a financial risk to the sustainability of the FPGC, and could increase patients survival and life quality.

## Background

The implementation of the SPSS in 2004 initially arose with the purpose of providing public health services to the population without social security in Mexico. This plan became the health insurance system that guarantees access to health services to everyone regardless of their employment or socioeconomic status. The SPSS, which operates through the Seguro Popular (SP-entity that operates as an insurer), covers a specific catalog of health interventions through the Catálogo Universal de Servicios de Salud (CAUSES-Universal Catalog of Health Services) and a high cost interventions package called Fondo de Protección contra Gastos Catastróficos (FPGC-Fund for Protection against Catastrophic Expenditure). In addition, the Seguro Médico Siglo XXI (SMSXXI-21st Century Medical Insurance) was created, and provides additional coverage of what would be considered in CAUSES and FPGC for children under 5 years old [1]. Among the main objectives of the SP was to offer health coverage for vulnerable population, besides reducing catastrophic and impoverishing expenditures. High cost diseases (cancers, lysosomal diseases, HIV, etc.) represented a significant financial burden for households that lacked access to health insurance, most of the population without access to health services suffering from a highcost disease did not receive medical attention. Therefore, the FPGC was created; the objective of this fund is to cover highcost diseases for population affiliated to the SP, and includes an explicit catalog of interventions. An important part of the FPGCs resources is spent on breast cancer treatment [2]. Breast cancer is globally classified among the main public health problems [3], the most frequent occurring in women; each year 1.38 million of new cases are detected and 458,000 deaths are caused by this disease. It is estimated that about half of cancer cases and 60$\%$ of the deaths are reported in developing countries [4]. According to data from the Pan American Health Organization (PAHO) in 2012, in Latin America and the Caribbean, 27$\%$ of new cancer cases and 15$\%$ of deaths caused by cancer are due to breast cancer. In North America, 30$\%$ of new cases and 15$\%$ of deaths caused by cancer in women are attributed to breast cancer [5]. In Mexico, breast cancer currently is ranking first in terms of incidence of neoplasms in women, accounting for 11.43$\%$ of all cancers. The most affected group is between 40 and 59 years old [6]. The number of breast cancer cases in Mexico was estimated at 20,444 and fatal cases were 5595 for the year 2012 [7]. There is a strong relation between age and mortality in breast cancer, the number of patients having this disease is significantly increasing from 30 to 64 years old. On the other hand, and for obvious reasons, mortality increases with age [8].

### The coverage of breast cancer on the FPGC

TThe FPGC has as objective to cover financially the attention of diseases of high specialty and high cost, which may endanger peoples lives and families heritage. Because of agreements signed with the 32 federal entities, it provides monetary resources through a trust. It operates through a group of health units providing health services, which are accredited to offer interventions covered by the FPGC. The coverage of breast cancer by the FPGC started in 2004. At the end of the fiscal year 2015, the catalog of the FPGC ended up with 61 interventions that cover nine groups of high cost diseases. According to the information of the Comisión Nacional de Protección Social en Salud (CNPSS-National Commission for Social Protection in Health) [2], from 2004 to 2015 the FPGC financed 1,082,805 served cases that represent $48,782.7 million pesos [9]. It is important to highlight that more than 50$$\%$$ of the FPGCs resources is spent in breast cancer treatments, intensive neonatal care, colon and rectum cancer, and a large proportion for financing antiretroviral drugs for HIV/AIDS care. In order to consider the attention cost of a disease as a catastrophic expenditure, it requires that the Consejo de Salubridad General (CSG-General Board of Health Standards) issues a protocol; such protocol highlights the process of clinical care given by medical experts in terms of disease care, as well as the treatment duration and necessary medicines. Within the portfolio of the FPGC medical services, there is a subset of diseases for which there is a maximum period of attention or maximum age covered. This group of diseases, which has a maximum period of attention with resources of the FPGC, represents a challenge for the SPSS as to fulfill the maximum period of attention; there is not a mechanism to ensure access to health care with resources from the SPSS for this specific population. One of the diseases that is in this situation is breast cancer: monitoring must be performed every 4–6 months for the first 5 years, as indicated by the care protocol issued by the CSG. On the other hand, the SPSS generates a tariff and sets the payment rate, which includes the cost for each care stage, the tariffs of each intervention in the catalog of the FPGC are revealed to service providers [2]. There is a debate in terms of the optimum monitoring period, for example, the monitoring recommended by the Instituto Nacional de Cancerología (National Institute of Cancerology) is 20 years, the main objective is to detect local, regional or systemic relapse and the presence of a second primary cancer. Thus, such monitoring should consider mammography and thorax teleray, as well as a biannual bone densitometry in postmenopausal women or those treated with aromatase inhibitors [6]. Recent studies show that the overall survival of the 202 women monitored in the first 3 years was 82$$\%$$ and the disease-free survival 63$$\%$$. Moreover, a second work with longer monitoring data showed an overall survival of 10 years of 60$$\%$$ and a disease-free survival of 22$$\%$$ [10]. In the research performed on similar women with breast cancer, that had clinical monitoring for more than 10 years, the survival rate was 69.0$$\%$$ in those with infiltrating ductal carcinoma and 84.0$$\%$$ of those affected with the classic lobular disease type. The latter proves that life expectancy is larger for those having classic lobular carcinoma receiving more clinical monitoring [11]. The international evidence shows that the recommended monitoring period varies between 5 and 25 years, depending the stage when breast cancer was detected, there are still differences in terms of monitoring resulting in an increase of patients survival. However, it is clear that the more monitoring there is for patients, the greater likelihood of timely detection of the disease recurrence. Given that the FPGC covers a clinical protocol for breast cancer monitoring in a defined interval of time (5 years), and due to the positive response to the treatment, there is a segment of women with higher survival rate as specified in the care protocols, which represents a gap in financing to ensure the continuity of follow-up and treatment for the covered population. Table 1 shows the general protocol for breast cancer treatment per state, diagnosis, treatment and follow-up.

**Table 1:** Protocol to treatment breast cancer. Source: Ministry of Health

I. Diagnosis	II. Treatment	III. Follow-up
Clinical examination	Surgery: conservative, mastectomy	Medical consultation and clinical examination
Self-exploration	Chemotherapy	Mammography
Image studies	Radiotherapy: brachytherapy, external radiotherapy	Gynecological follow-up in case of treatment with tamoxifen
Biopsy	Biological therapies	Bone densitometry in case of treatment with aromatase inhibitors
Immunohistochemistry	Endocrine therapy	
Tumor pathology		
Others		

The main objective of this research is to determine the financial impact and the financial risk quantification that would represent an increase on the follow-up for women with breast cancer that exceed monitoring time covered through the FPGC. It seeks to quantify the financial impact in short and medium terms, and financial sustainability of the FPGC, in order to monitor women with breast cancer that have persistence after covering the 5 year monitoring established in the protocol issued by CSG. The following section presents the sources of information and methods used for the analysis. Subsequently, the evolution of breast cancer cases and those covered by the FPGC are presented, the demographic structure of the population served is analyzed and the epidemiological profile is determined using a micro-simulation model to follow-up on a cohort of women. The probable cost scenarios are assessed in medium term, using the Monte Carlo simulation model and the financial impact is estimated through the Value-at-Risk (VaR) of the maximum expected expenditure of the monitoring expansion. In the last section, we presented the conclusions; financing alternatives are proposed to cope with the expected additional cost as a result of the estimated scenarios.

## Methods

We estimated the financial impact derivative from the extension of the monitoring period for breast cancer in women who require it at the end of the 5 year period covered by the protocol of current care, and which is financed by the FPGC. The estimate of sensitive population who are meant to receive attention, with the monitoring expansion, was carried out through a micro-simulation model following-up a cohort of women looked after by the SPSS for the period 2013–2026. The expected number of breast cancer cases was projected per year, taking into account the expected annual incidence rates and demographic change.

Subsequently, based on the simulations of the susceptible population, the costs of monitoring care for this population was estimated using the micro costing methodology, as well as the maximum expected cost in attention of a medium-term horizon using the Monte Carlo simulation. This method has the vantage of following a cohort of patients. The estimations were based on scenarios and trends of morbidity and mortality of patients covered by the SPSS in health units of the Ministry of Health. This can be limited because there is a group of patients who attend social security and private institutions, and a group of patients who has not received timely detection of cancer, underestimating the national reality. However, the perspective of the study focuses on knowing the financial sustainability of the expansion of the monitoring period exclusively for the SPSS, based on the best available information. estimates will allow knowing a future outlook of the disease and the possible costs scenarios of increasing the monitoring period, which should be an input for public policy decision-making. Estimations were made based on the historical information of population covered, cases attended, morbidity and mortality of the SP and the Ministry of Health for the 2007 to 2013 period. Based on the number of women covered by the SPSS, and taking into account the observed incidence in the Ministry of Health, the expected total breast cancer cases for the following years were estimated (see Eq. 1). In addition, the number of cases that were diagnosed in previous years was identified and that in 2013 were monitored. With the latter, the total number of served cases (prevalence) was obtained. It is important to remember that the SPSS includes in its FPGC service portfolio the attention of this intervention since 2007, which is why there is a record of the number of cases that will require monitoring in short term. The susceptible population was determined by performing a distribution of the average population from the Consejo Nacional de Población (CONAPO-National Population Council) regarding the population affiliated to the SPSS per age group in 2013.

1 $$\begin{aligned} C_{i,e}&=\left[ \sum _{i,e}Io_{e}(Ps_{i,e}+\sum _{j,e}Cs_{j,e})\right]; \quad i=2013,\ldots,2026\nonumber \\ e&=0,\ldots,100; \quad j=2007,\ldots ,2013 \end{aligned}$$

where $C_{i,e}$ are the number of cases per year and age.

$Io_e=\frac{\sum _{k,e}\frac{Cn_{k,e}}{Pr_{k,e}}}{n}; k=2007,\ldots ,2013;e=0,\ldots ,100$ are the average incidences observed per age; $C_{n},$ are the new cases and $P_{r}$, is the population at risk of contracting the disease. $Ps_{i,e}=PC_{i,e}(PA_{l,e}); i=2013,\ldots ,2026$; $k=0,\ldots,100$; $l=2013,$ is the susceptible population per year and age; $PC_{i,e},$ is the average population of CONAPO per year and age; $PA_{l,e},$ is the distribution of the population affiliated to the SPSS per year and age in 2013, with $e=0,\ldots,100$ and $l=2013$. $Cs_{j,e}$ is the population diagnosed in previous years per age, and that in 2013 were monitored during the treatment, with $j=2007,\ldots , 2013.$

To evaluate the survival, 14 monitoring cycles were estimated based on the historical fatality rate, considering a time horizon of 2013–2026. For each cycle, the number of new identified cases was taken into account, based on the estimated incidence, and the corresponding fatality rate was applied per age in order to obtain the expected deaths. Such estimations were performed in order to determine the survival after a year of treatment and 5 years of monitoring covered by the SPSS (see Eq. 2),

2 $$\begin{aligned} S_{i,e}=\sum _{i,e}\left[ C_{i,e}-C_{i,e}(L_e)\right] ; \quad i=2013,\ldots ,2026;\;\; e=0,\ldots ,100 \nonumber \\ \end{aligned}$$

where $S_{i,e}$ are surviving cases per year and age; $C_{i,e}$ is the number of cases per year and age.

$L_e=\frac{\sum _{k,e}\frac{M_{k,e}}{D_{k,e}}}{n}$;$k=2007,\ldots,2013;e=0,\ldots,100,$ is the average fatality rate observed per age; $M_{k,e}$, are deaths per year and age; $D_{k,e}$, are the diagnosed cases per year and age; and *n* is the number of years considered.

Taking into consideration an average duration of a 1-year treatment, the last monitoring financed by the SPSS for women diagnosed in 2013 and that would be entitled to, according to the care protocol, would be 2018. In order to establish public policy recommendations, the decision was to start the analysis in 2013 and to follow-up during 3 periods of federal government (18 years), it allows assessing the relevance of contemplating this situation in the budgets of the following administrations and link the financial impact directly with the budget. The estimated cost for expanding the monitoring period covered by the SPSS was obtained as the result of the number of cases requiring monitoring multiplied by the cost of monitoring (see Eq. 3).

3 $$\begin{aligned} C_i=Cs_i*Ks_i;\quad i=2013,\ldots ,2026 \end{aligned}$$

where $C_{i}$ the cost of expanding the period of monitoring per year; $CS_{i}$ cases requiring monitoring per year are cases that exceed the period of coverage currently provided by the SPSS; $Ks_i=M_i+AC_i$ is the cost of monitoring per year, which is updated every year with an inflation of 3%; $M_{i}$ is the cost of medicines (see Appendix 1); $AC_{i}$ is the cost of clinical analysis (see Appendix 2).

The costing methodology is based on a micro-costing approach, it is important to emphasize that in case of the FPGC the finance coverage of interventions is made per event, for which an integral cost of care considered, for more details about the costing methodology see [12]. For evaluating the financial impact generated by the additional cost of extending the monitoring period of the breast cancer treatment in women who exceed the period financed by the SPSS a maximum expected cost was estimated, as a modified version of VaR through the Monte Carlo method by performing 5000 simulations, the estimation of VaR was performed with a confidence level of 95$\%$ [13–16].

4 $$\begin{aligned} \int _{V_c}^{+\infty }r(s)ds=\alpha \end{aligned}$$

where *r* is the monitoring cost; $\alpha$ is the significance level; $V_{c}$ is the cut-off point based on the established confidence level $(1-\alpha ).$

It is important to mention that in this case for implementing the VaR, the objective is to determine the maximum expected cost of monitoring patients with breast cancer, so the integration interval is inverted with respect to a traditional VaR estimation. In this case we seek to estimate the maximum expected cost, so we take the right tail of the distribution of possible monitoring cost values. The cut-off value is the value for which there is a probability that the monitoring cost ends on top of this, which corresponds to finding the maximum cost ensuring that only 5 out of 100 iterations may end in this value ($\alpha =0.05$) (see Eq. 5).

5 $$\begin{aligned} P\left[ c(W)\ge V_c\right] =(1-\alpha ) \end{aligned}$$

The considered risk factors for the Monte Carlo simulation and their probability distributions were as follows (Table 2).

**Table 2:** Risk factors for the Monte Carlo simulation. Source: The Authors

Risk factor	Distribution
Incidence	*ln*N($\mu$,$\sigma ^{2}$)
Fatality	*ln*N($\mu$,$\sigma ^{2}$)
Costs	*ln*N($\mu$,$\sigma ^{2}$)

The variable of interest for estimating the VaR is the cost per increase in the monitoring period covered by the SPSS; for this, the evolution of such cost was modeled through a stochastic lognormal process (see Eq. 6).

6 $$\begin{aligned} C_i=C_{o}e^{\left( \mu -\frac{\sigma ^2}{2}\right) \Delta _i+\sigma \sqrt{\Delta _i\xi }} \end{aligned}$$

where $$\xi \sim N(0,1); \Delta _i=i-i_0.$$

Three scenarios were estimated (base, pessimistic and optimistic) for generating the VaR. For the base scenario, the parameters taken into consideration, were incidence, mortality and costs observed for the pessimistic and optimistic scenarios expected values of such parameters were generated using a normal distribution standard. The pessimistic scenario considered a range of expected increase of the observed incidence of up to 10$\%$, also a cost variation was simulated in the same proportion. This variation of up to 10$\%$ in incidences and costs was considered taking as reference the historical behavior of such parameters [2]. The optimistic scenario considered a decrease in incidence and costs, allowing to evaluate a floor for the expected total cost taking into consideration that the expected incidence in the coming years decreases, as a result of the breast cancer prevention campaigns. Even when it is an unlikely scenario, it is important to have a range of maximum cost expected to be able to make decisions regarding the consideration of future budget. In terms of costs, there is the possibility of maintaining a policy of promoting generic drugs, as some of the molecules used in the breast cancer treatment will lose their patent in coming years, so it would be feasible to reduce the monitoring cost. In addition, there is a policy of containment and price reduction for medicines with patent in force or single source, through the negotiation of public procurement prices and consolidated purchases, which will make possible that in short term treatment costs will not increase. Regarding the sources of information, registered breast cancer cases (incidence) in the Ministry of Health were used, which were financed by the FPGCs portfolio of services and estimated costs for monitoring treated patients. At the same time, the specific cause mortality was used for population treated in the Ministry of Health, and CONAPO projections of average population for the period 2013–2050 were utilized in order to use the demographic structure of population covered by the SPSS in a long term horizon. The estimations presented consider current information regarding the care protocol and the current FPGC coverage. The objective is to evaluate if increasing monitoring is financially viable, in case it allows to detect relapses, their attention and treatment would be financed as a new case. The financial impact of possible relapse requires additional estimates of the sustainability of the FPGC, taking into account the evolution of all diseases covered in this fund.

## Results

### Evolution of breast cancer cases covered by the FPGC

**Fig. 1:**
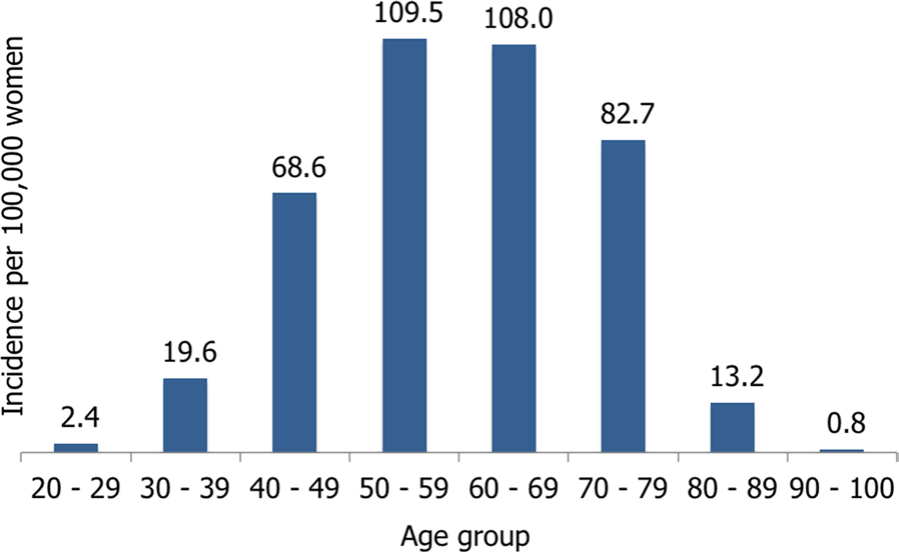
Average incidence of breast cancer per age group for population covered by the SPSS, 2013, (per every 100,000 women) (Source: The Authors)

The funding of the SPSS for breast cancer is performed per care phase and afterwards monitoring, this being post-treatment (treatment usually lasts a year). Such phase has a maximum of five annual events once the treatment is concluded, this being the main point of analysis in this research. Taking into consideration an average duration of a year, women diagnosed during 2013, in 2018 will have the last monitored year financed by the SPSS, according to the attention protocol issued by the CSG. Based on reported information by the Ministry of Health, the incidence observed in women affiliated to the SPSS per age group from 20 years and on was generated. Data shows that the population group in which there are more diagnosed cases is in the interval between 50 and 69 years old, which together account for 53.9$\%$ of the total affected population, and in groups of 40–79 years old represents 91.5$\%$ of the total number of cases (Fig. 1).

**Fig. 2:**
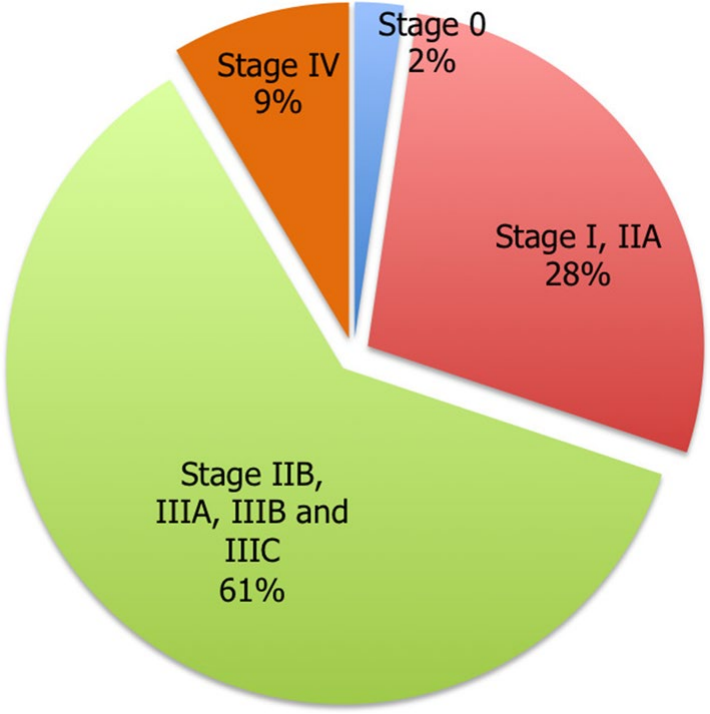
Distribution of the diagnosed population with breast cancer per stage (Source: The Authors)

According to information registered in 2013, out of the cases diagnosed with breast cancer treated by the SPSS, more than half of the population that was diagnosed in the group of 40–79 years old were classified in an advanced stage of the disease. In stage I and IIA recorded 28$\%$ cases; in stages II B and III that concentrate 61$\%$ of the cases, the treatment includes radiation treatments (all sessions that the patient requires), cosmetic breast surgery and reconstruction (without a prosthesis), chemotherapy and monoclonal antibodies; in stage IV 9$\%$ was diagnosed, which impacts on a more aggressive and expensive treatment (Fig. 2).

**Fig. 3:**
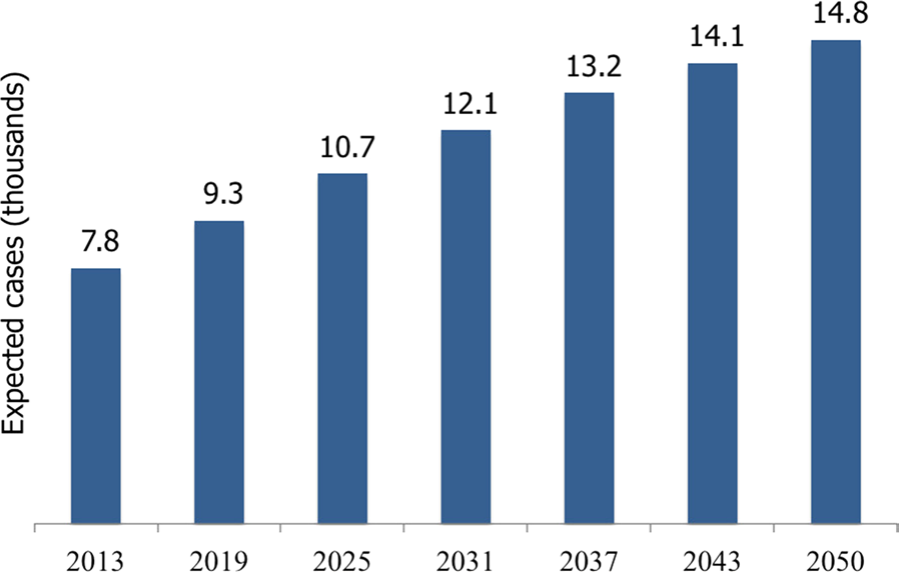
Evolution of new expected cases of breast cancer in SPSS 2013–2050 (1000 cases) (Source: The Authors)

Taking into consideration data of the Ministry of Health on population affiliated and diagnosed, and average population of CONAPO, the number of expected breast cancer cases was projected for the period 2013–2050 (Fig. 3). There has been an average annual increase of 1.74$\%$ in the period, from 2014 there has been an increase higher than population growth, this is due to aging of the population diagnosed in previous years and, therefore, changes of cases observed between age groups. Annual cases per age between 20 and 100 years were estimated. As mentioned above, the bulk of attention occurs in women between 50 and 69 years old, therefore, as time goes by the risk group is increasing.

### Survival of served population

**Fig. 4:**
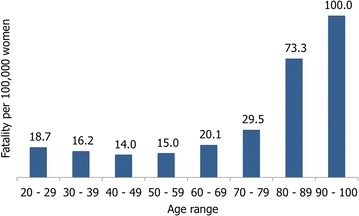
Average breast cancer fatality per age group for covered population by the SPSS, 2013 (per every 100,000 breast cancer patients) (Source: The Authors)

IIn the statistics of the Ministry of Health, the survival of population with breast cancer is not registered. Therefore, based on information of registered deaths of population affiliated to the SPSS and the total number of diagnoses of 2013, the mortality rate per age group was estimated. An overall fatality rate was estimated, because the available data does not allow to link cases according to the diagnostic phase with the patients outcome. Women between 20 and 29 years presented a fatality rate of 18.7 deaths per 100,000 diagnosed, women over the age of 60 years presented greater fatality rates as expected (Fig. 4).

From the fatality rate, was determined the number of probable cases that will continue to demand treatment for monitoring after 5 years financed by the SPSS. It is assumed that as the number of monitoring years for ill population increases, the probability of increasing their survival is greater, as several studies pointed out [17]. To evaluate the survival, 14 cycles were estimated based on the fatality rate for the 2013–2026 period. When generating each one of the cycles, new cases determined based on the estimated incidence were taken into account and the corresponding fatality rate was applied per age in order to obtain the expected deaths. These estimates were conducted for each one of the analyzed scenarios, in order to determine the survival rate after 1 year of treatment and the 5 years of monitoring covered by the SPSS. The number of women diagnosed in 2013 that survive after the year 2018 was determined and that by 2019 may be vulnerable to requiring additional monitoring than what was covered, and so on until reaching the cycle 14 with total cases per each cycle (see Table 3).

**Table 3:** Number of cases subject to monitoring for 14 cycles. Source: The Authors

Cycle/year	2013	2014	2015	2016	2017	2018	2019	2020	2021	2022	2023	2024	2025	2026	2027	2028	2029	2030
1	2531	2095	1732	1429	1177	968	794	649	530	431	349	282	227	182	145	115	91	72
2		2605	2156	1782	1470	1211	995	815	667	544	442	358	289	233	186	149	118	93
3			2680	2217	1832	1511	1244	1021	837	684	557	453	367	296	238	191	152	120
4				2754	2277	1881	1551	1276	1048	858	701	571	464	375	303	243	195	155
5					2828	2337	1930	1591	1308	1073	879	717	584	474	384	309	249	199
6						2901	2397	1979	1630	1340	1099	899	734	597	484	392	316	253
7							2973	2455	2026	1668	1371	1123	919	749	610	494	399	322
8								3044	2513	2072	1705	1401	1147	938	765	622	504	407
9									3114	2569	2118	1742	1430	1171	957	779	633	513
10										3182	2624	2162	1777	1458	1193	974	793	644
11											3249	2678	2205	1812	1486	1215	991	807
12												3313	2730	2247	1845	1512	1236	1008
13													3377	2780	2287	1877	1537	1256
14														3438	2829	2325	1907	1562
Total	2531	4700	6567	8182	9584	10,808	11,883	12,830	13,671	14,421	15,094	15,700	16,250	16,750	13,711	11,197	9121	7410

**Fig. 5:**
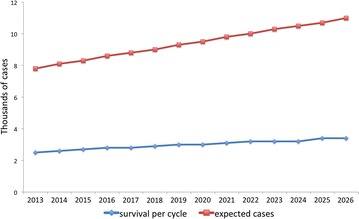
Expected cases and survival per cycle, 2013–2026 (Source: The Authors)

Cycle 1 (2013) corresponds to the number of women diagnosed in 2013 that after receiving a year of treatment and 5 years of monitoring by 2019 have survived, giving as a result 2531 cases. The number of diagnosed cases was 7841, which allows to observe that the survival rate is approximately 30$\%$, this percentage being similar to the rest of the generated cycles, and is consistent with data reported in other studies. For 2026 (14 cycles) 11,000 new cases and 17,000 cases in monitoring phase are expected, this is taking into account surviving population from previous years, and contemplating that they exceeded 5 years of monitoring currently financed (Fig. 5).

### Financial impact through the estimation of value of risk with the Monte Carlo method

**Fig. 6:**
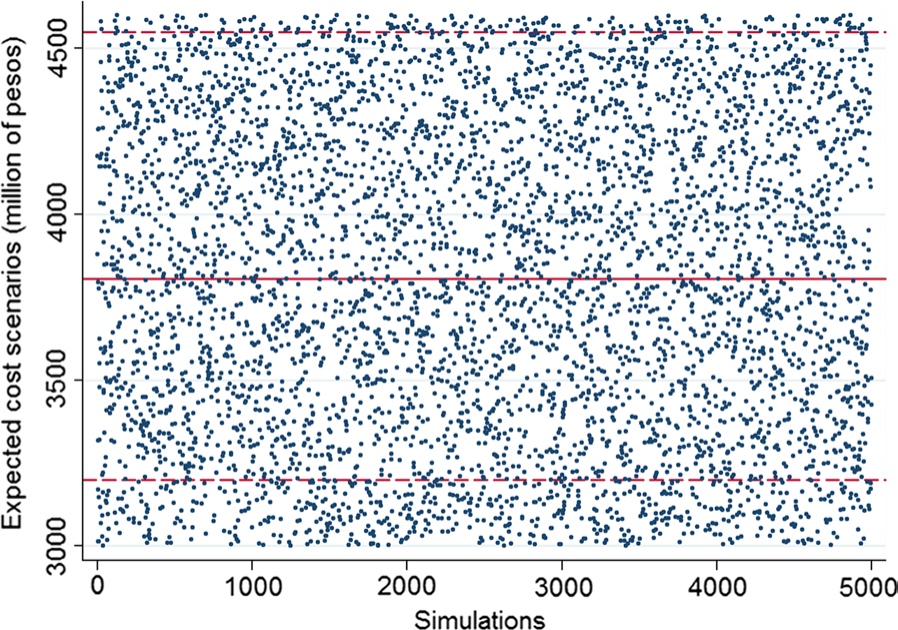
Different cost scenarios simulated by Monte Carlo method. Source: The Authors. Dotted line represents the average cost in optimistic and pessimistic scenario, continuous line represents average cost of base scenario

The scenarios estimated through Monte Carlo simulation show the range of estimated cost. For evaluating the financial impact, three scenarios were analyzed (base, pessimistic and optimistic) for estimating the VaR (maximum expected cost). Through the simulation of random variables for the parameters used in each scenario and taking into account the cycles of attention (three 6-year presidential periods) the VaR generated at 95$\%$ confidence was obtained, based on the simulation of 5.000 iterations. Figure 6 shows the map of estimated values for the simulated data.

**Fig. 7:**
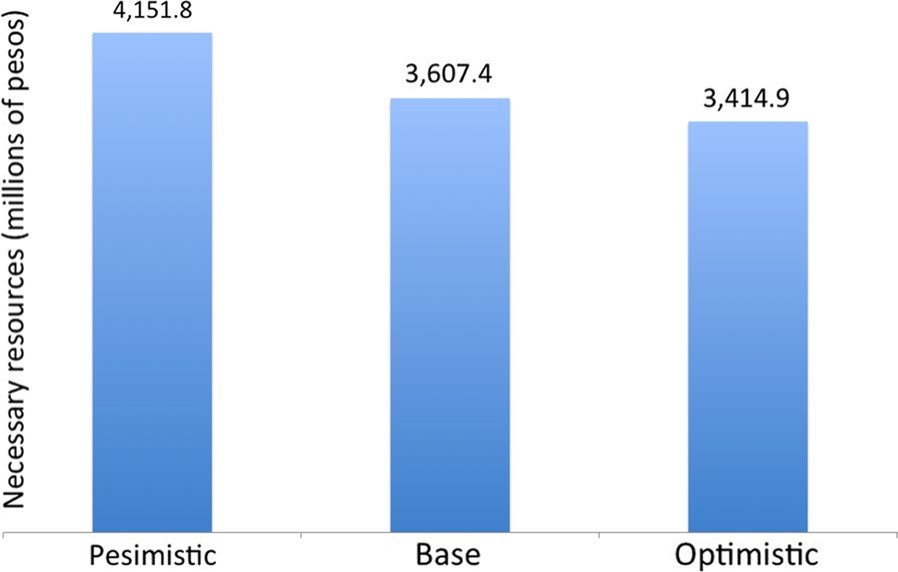
Maximum expected cost to be financed for expanding the monitoring period (Source: The Authors)

The maximum expected cost estimated through the calculation of VaR for the scenarios raised, allows to know the amount of resources that should be contemplated to cover the monitoring of women diagnosed with breast cancer from the year 2013, and that exceed the monitoring period of 5 years after their treatment, contemplated in the care protocol in force covered by the SPSS (Fig. 7).

The base scenario, contemplates the results obtained by using the observed parameters of incidence, mortality and costs. The maximum cost estimated for the number of vulnerable cases to require monitoring for 14 cycles represents a cumulative amount of $3607.40 million pesos and an average annual cost of $200.41 million pesos of 2013–2026. Taking into account that only monitoring will be covered to a minimum of 10 years for patients diagnosed until 2015 and a maximum of 23 years for patients diagnosed in 2013. It is necessary to have a reference to analyze the maximum expected cost to extend the years of monitoring, in this case, the available resources having the FPGC should be contemplated. According to the results report of the SPSS of 2013, the FPGC trust has an availability of resources of $4487 million pesos (Table 4).

**Table 4:** Availability of the FPGC resources at the end of the 2007–2013 period (million pesos). Source: The Authors

Concept/year	2007	2008	2009	2010	2011	2012	2013
Availability	2282.44	3287.23	6319.60	5467.98	7628.57	10,388.90	4487.40
Contributions	4035.77	5687.10	7057.30	7284.70	8649.20	10,872.80	11,674.90
Commitments	2520.94	2654.77	7908.90	5124.11	5888.87	16,774.00	9418.80
Availability at the end of the financial year	3797.27	6319.56	5468.00	7628.57	10,388.90	4487.70	6743.50

Considering the three scenarios for the risk factors and a monitoring period of 14 cycles (18 years) it would be feasible to finance the expected additional cost, only if the behavior of the rest of the interventions of the FPGC maintain their current behavior. However, the fact of covering up to 10 more years of monitoring impact positively on the quality of life of thousands of women. The average annual availability of resources is larger than $200.41 million pesos that would be needed annually to cover monitoring of more than 200,000 women throughout the estimated period, which means that extending the monitoring period would be affordable in the following three presidential periods. In addition to the latter, it is necessary to take into consideration that this mount would be amortized in 18 years which should be kept in mind prior to the completion of the annual budget. The expansion of monitoring to a minimum of 10 years and a maximum of 23 years would represent an additional average budget of $200.41 million pesos, guaranteed for the next 18 years, in the base scenario (optimistic scenario that amount would decrease to $189.71 million pesos a year and in the pessimistic scenario it would increase up to $230.66 million pesos). On the other hand, the annual average budget of $200.41 million pesos represents approximately 9.1$$\%$$ of the annual expenditure spent in breast cancer care and 3.0$$\%$$ of the availability of FPGC resources in 2013.

## Discussion

Breast cancer care is a priority topic for Mexico, due to the impact this disease has on womens health, household economy and finance of public institutions providing health services. It is recommended that the monitoring period is extended with the purpose of offering a better quality of life and a greater probability of survival for women who suffer from the disease. This strategy would complement the early detection campaigns that have been implemented in recent years. The implementation of this measure is affected by economic factors because the FPGC has limited resources, management procedures to modify the clinical protocol, and the authorization of the use of resources in the extension of actions in an intervention, see Tables 5 and 6 in Appendix. These elements should be considered into an adequate and financially sustainable implementation. The decision-making process must be based on more solid foundations, and being able to quantify the financial impact through scenarios. Using the micro-simulation model and the estimation of the maximum expected expenditure, the financial feasibility of extending the monitoring period for patients with breast cancer that exceeded the period currently financed by the SPSS (for a minimum of 10 years and up to a maximum of 23 years) was evaluated. This approach seeks to bond the need of resources with the budgetary cycles linked to the presidential periods, which will raise awareness of the need of resources of each administration and to justify the generation of reserves to cover the additional cost of extending the monitoring period. Based on the obtained results, the following recommendations are made: increasing monitoring in breast cancer patients that have exceeded the attention period currently financed up to a minimum of 10 years after the first year of treatment. Consider the annual budget for breast cancer care in an annual average amount of $200.41 million pesos to guarantee a minimum of patients to be served with the extended monitoring period. Foster the use of generic medicines in clinical protocols, on feasible cases, to generate economies. Evaluate the rest of interventions contemplating a maximum care period to prioritize the increase of such periods in cost-effective terms. Contemplate the possibility of establishing a copayment to ensure that patients with breast cancer that exceed the time of attention currently financed can continue in monitoring and treatment for a longer period according with international clinical standards. The implementation of the SPSS and the FPGC has generated positive results in terms of expanding the coverage and reducing catastrophic expenditure. So the next step is to achieve services with more quality and thus a healthier population that contributes to social and economic development of the country. This research presents possible scenarios of survival and monitoring costs for patients covered by the SPSS, in no way represents a sectorial view of the Mexican Health System, even though the SPSS serves approximately 50$$\%$$ of the population. The results have no external validity for any institution other than the SPSS, since the main objective is to evaluate the financial sustainability of increasing the monitoring period for the population covered by the SPSS. The results generated are based on the information available to the SPSS, in addition to considering the care protocol with which the disease is currently covered, so if there were any change in the care protocol it would be necessary to update the presented estimations.

## Conclusions

Thanks to the implementation of the FPGC, the SPSS has accomplished its objective of reducing the catastrophic and impoverishing expenditures, yet there still are future opportunity areas. This policy should be accompanied at all times by the homogenization of medical services quality, provided by highly qualified staff, with standardized care protocols and clinical practice guidelines for each intervention that supports the care provided. Financing interventions included in the FPGC represents a big challenge because the necessary resources depend on demographic, epidemiological and financial factors. Hence, the importance of conducting an in-depth analysis to manage the potential risks for the SPSS of including interventions or modifications to existing protocols. An important segment of the resources of the FPGC is destined for breast cancer treatment, because it was presented as the first cause of death for women between 40 and 59 years old. There is a debate about the need of extending the monitoring period as a measure to increase the probability of survival and life quality of patients, prevent local, regional or systemic relapse and the presence of a second primary tumor. Due to the fact that including breast cancer in the FPGC was in 2007, from 2012 cases of patients who exceed the monitoring financed period are happening and will be left without coverage, that is the reason why it is essential to evaluate the financial impact for the SPSS to extend that period. Currently, the SPSS does not have a mechanism that guarantees continuity and access to medical care for population that exceeds the maximum period of financed care with resources from the SPSS. The results obtained in this work indicate that the population of women diagnosed with breast cancer presents a survival rate after 5 years of monitoring to approximately 30$$\%$$ of the diagnosed cases. However, the incidence rate is not proportional to the fatality rate, which can be produced by the little monitoring given to patients. It is important to emphasize that the FPGC finances the interventions per event, so that a patient who was treated for breast cancer once her treatment was completed and the monitoring period contemplated in the protocol, lacks funding to continue with the monitor in later years. The estimation of three scenarios of maximum expected cost (base, pessimistic and optimistic) through a micro-simulation and the Monte Carlo method, allowed to determine the necessary financial resources to provide monitoring from 10 to 23 years to women diagnosed with breast cancer at a confidence level of 95$$\%$$, which rises up to $3607.40 million pesos for every period in the base scenario ($4151.79 million pesos in the pessimistic scenario and $3414.85 million pesos in the optimistic scenario). Such expenditure would be amortized in the following 18 years which would represent approximately an annual increase of 9.1$$\%$$ of the resources destined to the disease care and would use the 3.0$$\%$$ of the availability of resources of the FPGC taking into consideration the base scenario estimations (2.8$$\%$$ in the pessimistic scenario and 3.4$$\%$$ in the optimistic scenario). According to estimates made of the additional cost for extending the monitoring period of breast cancer cases would be financially viable taking into consideration the available resources of the FPGC, considering that there are no significant changes in the epidemiology and costs of the rest of the interventions covered by the FPGC.

## Appendix 1

**Table 5:** Drugs used for the follow-up. Source: Social Protection System in Health (SPSS)

Tamoxifen, Citrate of	
Exemestane 25 mg tablet	
Anastrozole 1 mg tablet	
Zoledronic acid monohydrate 4	
Letrozole	

## Appendix 2

**Table 6:** Clinical analysis used for the follow up. Source: Social Protection System in Health (SPSS)

Carcinoembryonic antigen (ACE)	
CA 15.3 antigen	
CA 27–29 antigen	
Biometry or complete cytology	
Serum calcium	
Immunofluorescence	
Lipid profile (triglycerides, total cholesterol, LDL and HDL)	
Liver function tests	
Blood chemistry 3 elements	
Densitometry 1 region	
Bone gammagram with technetium	
Bilateral mammography	
PET-CT-FDG	
Radiography 1 projection	
MRI of the abdomen and pelvis	
Computerized axial tomography without contrast of each one of the regions of the body	
Liver ultrasound	
Bilateral bilateral ultrasound	
